# Association of reduced peak left atrial strain with supraventricular arrhythmia in adults with congenital heart disease

**DOI:** 10.1007/s10554-024-03205-9

**Published:** 2024-08-16

**Authors:** Clément Nussbaumer, Markus Schwerzmann, Elena Elchinova, Eleni Goulouti, Daniel Tobler, Matthias Greutmann, Kerstin Wustmann, Andrea Papa, Fabienne Schwitz

**Affiliations:** 1 Department of Cardiology, Inselspital, Bern University Hospital, University of Bern, Bern, Switzerland; 2 Division of Cardiology, University of Ottawa Heart Institute, Ottawa, Ontario Canada; 3 Department of Cardiology, Basel University Hospital, Basel, Switzerland; 4 Department of Cardiology, Zurich University Hospital, Zurich, Switzerland; 5 Department of Pediatric Cardiology and Congenital Heart Disease, German Heart Center Munich, Munich, Germany; 6 Department of Cardiology, Inselspital, Center for Congenital Heart Disease, Bern University Hospital, Freiburgstrasse 18, Bern, 3010 Switzerland

**Keywords:** Adult with congenital heart disease, Atrial arrhythmia, Left atrial strain, Speckle tracking echocardiography

## Abstract

Atrial arrhythmias are an important cause of morbidity and mortality in adults with congenital heart disease (ACHD). In acquired heart disease, the left atrial (LA) strain has been shown to predict supraventricular tachyarrhythmias (SVT). This study aimed to investigate whether reduced LA strain is associated with SVT in ACHD patients. This retrospective, single-center cohort study collected baseline clinical and echocardiographic data of 206 ACHD patients (157 left heart defect, 49 right heart defect). Patients with sinus rhythm at baseline and a 5-year follow-up (median age 29, IQR 22–41 years) were included. Diagnosis of sustained SVT was determined from clinical reports during the follow-up period. New or recurrent sustained SVT occurred in 16 patients (7.8%, median follow-up of 6.2 years). Patients who developed SVT were older, more likely to have diastolic dysfunction, and had larger LA dimensions, left ventricular mass, and a lower peak LA longitudinal strain (PALS). Lower PALS was associated with higher risk of SVT in patients with left and right heart defects. Patients in the lowest quartile for PALS had a 15.9-fold higher hazard ratio of SVT (95% confidence interval, 4.5 to 56.0, *p* < 0.001) in comparison with the top three quartiles. PALS provides information about the occurrence of SVT in the ACHD population. Including measurement of LA strain in the follow-up of these patients may allow to better identify patients at risk of future atrial arrhythmias.

**Graphical abstract:**

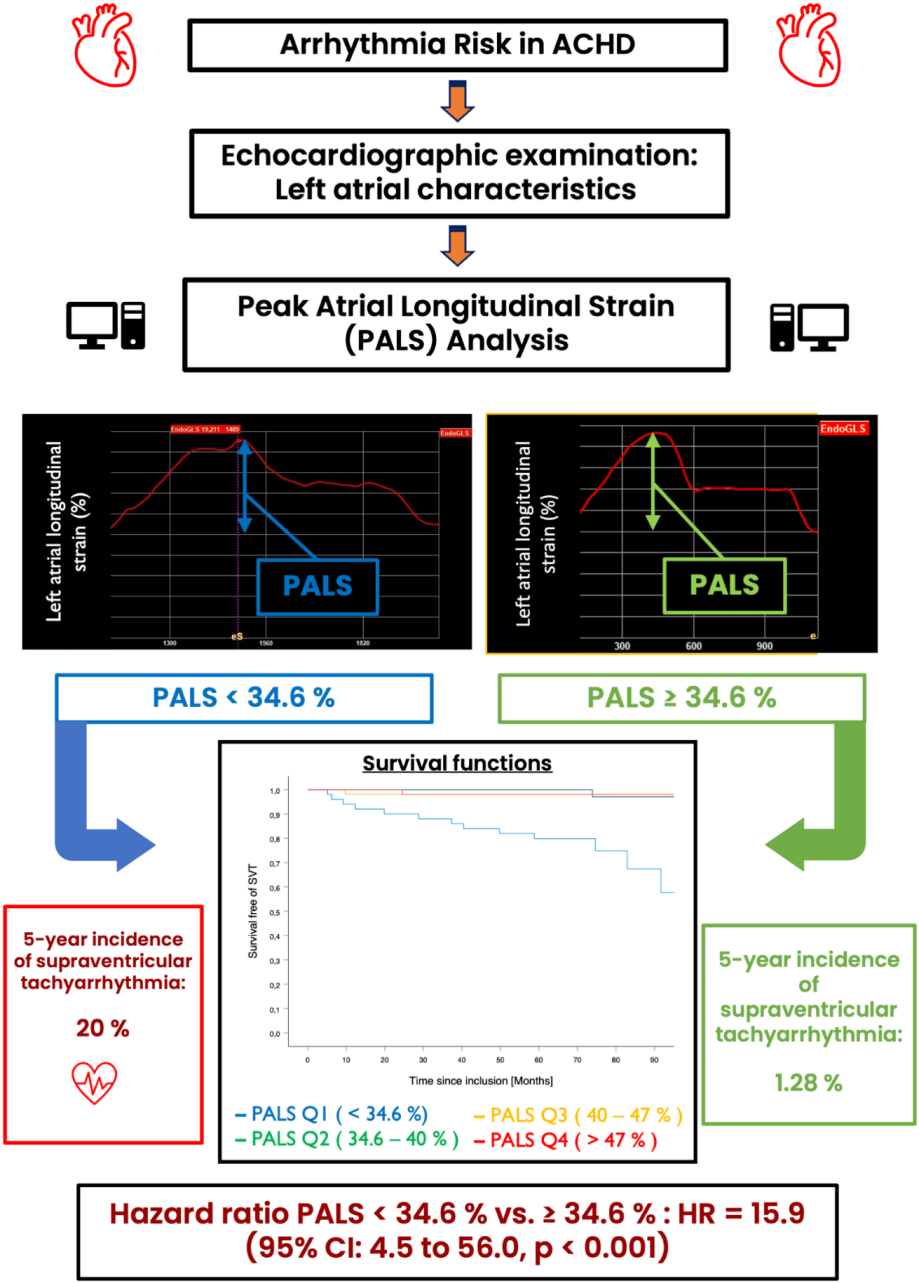

## Introduction

Due to significant advances in medical care over the past decades, more than 90% of patients with congenital heart disease now reach adulthood [1]. The overall median age expectancy of the population of adults with congenital heart disease (ACHD) has also greatly increased throughout this period [2, 3]. For many patients, this implies a longer exposure to hemodynamic alterations secondary to residual lesions and sequelae from previous cardiac interventions. As a result, late complications are more likely to occur, arrhythmias being the most frequent [4, 5]. In a previous study, Bouchardy et al. estimated the 20-year risk of developing atrial arrhythmia to mount up to 38% for a 50-year-old patient with a congenital heart defect [6]. Atrial arrhythmias are associated with increased mortality and impaired quality of life. Identifying patients at high risk for developing these arrhythmias could impact their medical follow-up and reduce the morbidity burden of arrhythmias [7, 8].

Clinical and echocardiographic parameters, such as age, complexity of congenital heart defect, previous heart surgery or left atrial (LA) volume, are weak predictors of atrial arrhythmias [8, 9]. With the advent of atrial speckle tracking echocardiography, it is possible to assess left atrial function on the tissue level, in addition to functional measures based on changes in left atrial size. Peak atrial longitudinal strain (PALS) and peak atrial contraction strain (PACS) are considered surrogates for the reservoir and booster atrial function. Recent studies have demonstrated the independent and incremental prognostic value of left atrial strain analysis for the development of supraventricular arrhythmias in patients with acquired heart disease [11–13]. In these studies, reduced PALS and PACS values combined with clinical and other echocardiographic parameters, were able to identify patients at high risk of developing supraventricular arrhythmias. Our study aimed to assess the association between reduced LA strain and supraventricular tachyarrhythmias (SVT) in patients with congenital heart disease, including left and right heart lesions.

## Methods

### Patients

In this retrospective single-center cohort study, ACHD patients from the University Hospital of Bern (Inselspital) participating in the Swiss Adult Congenital HEart disease Registry (SACHER) were identified. The inclusion criteria encompassed patients with working diagnosis of aortic stenosis, coarctation of the aorta, or Fallot physiology. The rationale for selecting these defects was (i) include frequent cardiac defects to have a sufficiently large patient cohort, (ii) limit the heterogeneity of the congenital defects, (iii) include also patients with mainly right heart disease. Data collection and analysis was conducted between November 2021 and March 2022. A total of 297 patients diagnosed with the mentioned conditions and undergoing clinical visits from January 2011 to December 2016 were documented, allowing for a minimal follow-up duration of 5 years in all patients.

Exclusion criteria were age < 18 years at baseline echocardiographic examination, absence of sinus rhythm at baseline, no follow-up at least five years after the index consultation, inadequate echocardiographic images for off-line strain analysis (including patients with foreshortening of the left atrial cavity in the 2 chamber view) and more than mild mitral stenosis or mitral regurgitation. The study protocol has been approved by the ethics committee of Bern (KEK) and all the patients gave their consent, as part of SACHER. The study complied with the Declaration of Helsinki.

### Echocardiography and strain analysis

Patients underwent echocardiographic examination as part of their routine cardiologic outpatient visit. Echocardiographic imaging was performed using standard equipment (Philips IE 40). Conventional and tissue doppler analysis was carried out during acquisition, according to the EACVI textbook of echocardiography [14]. LA volume and function were retrospectively assessed by one observer (CN). Left atrial volumes were acquired using the modified biplane method of disks and indexed by body surface area. We excluded left atrial appendage and pulmonary veins for LA volume measurements. LA emptying fraction (global function) was calculated as [(LA maximal volume – LA minimal volume) / LA maximal volume] x 100%, and LA expansion index (reservoir function) as [(LA maximal volume – LA minimal volume) / LA minimal volume] x 100%. LA enlargement was defined as a left atrial volume index (LAVI) greater than 34 ml/m^2^, whereas left ventricular hypertrophy was defined as a left ventricular mass index (LVMI) greater than 115 g/m^2^ in men and greater than 95 g/m^2^ in women, according to current guidelines [15].

**Fig. 1:**
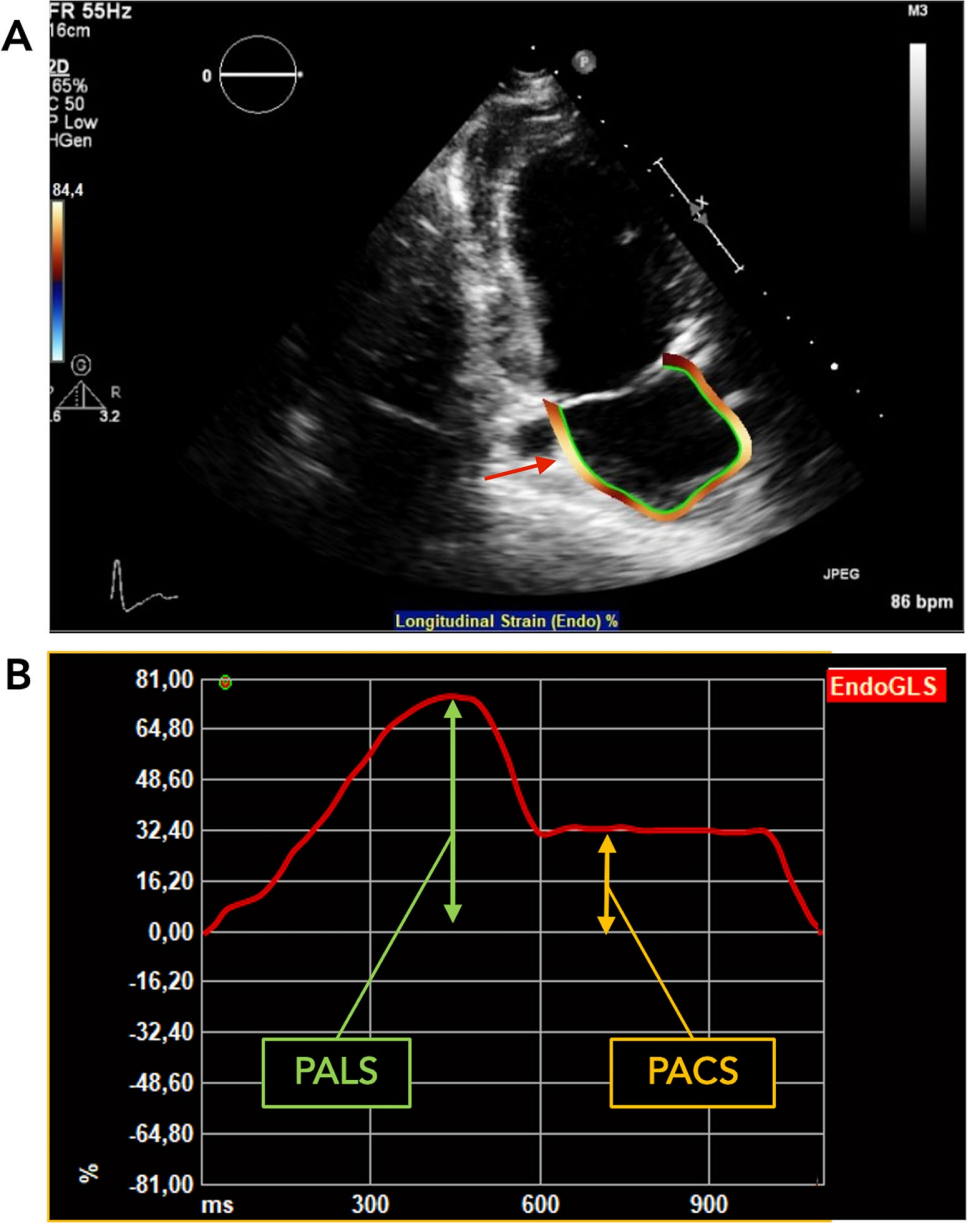
This figure shows left atrial strain analysis using speckle tracking echocardiography in 2-chamber view. (**A**) End-systolic automatically selected left atrial contour (red arrow). (**B**) LA strain profile (red line) of an ACHD patient using speckle tracking echocardiography during a complete cardiac cycle (end‑diastole to end‑diastole or R-R gated). PALS was calculated as the difference between peak and minimal strain values (green arrows). PACS represents the absolute value of the difference between minimal and plateau-phase strain values (yellow arrows)

Left atrial two-dimensional strain was measured using TOMTEC ad-hoc module with a speckle tracking method (2D Cardiac performance analysis 1.4) in the apical 2-chamber view. Temporal resolution ranged between 50 and 110 frames per second. We used the end-diastole – based on the electrocardiographic (ECG) R wave – to define the reference frame of zero strain [16]. LA contour at end-diastole and end-systole was defined automatically and then adjusted manually if not optimally fitted. The same method was used for patient with atrial patch repair. PALS was calculated as the difference between peak and minimal strain value. PACS was determined as the absolute value of the difference between the minimal strain value and the plateau phase strain value. The acquisition and measurement of LA strain are illustrated in Fig. 1.

### Clinical data, outcome, and follow-up

Clinical data and magnetic resonance imaging right ventricle baseline values were collected from patients’ clinical charts. The primary outcome was defined as new or recurrent sustained SVT during the follow-up period. This includes the recurrence of atrial arrhythmias after a symptom-free interval of at least six months following cardioversion or catheter ablation in patients with previous arrhythmias. SVT diagnosis, including atrial fibrillation, intra-atrial re-entry tachycardia, and focal atrial tachycardia, was based on documentation with either standard 12-lead ECG or with Holter ECG.

### Sample size calculation

Based on the results of Bouchardy et al., we assumed an SVT incidence of 4% in our study population [6]. Similar to the prognostic value of PALS for atrial arrhythmia in acquired heart disease, we hypothesized a hazard ratio of 6 for SVTs in patients with the lowest quartile of PALS compared to the patients in the highest quartile. Based on these assumptions a sample size of 240 patients was necessary to test our hypothesis. To minimize information bias, we measured LA echocardiographic parameters blinded to the patient’s clinical history and 5-year follow-up data.

### Statistical analysis

Categorical variables are presented as numbers and frequencies, and continuous variables as mean ± SD or median (IQR), depending on their distribution. To compare continuous variables among groups, we used one of the following tests depending on the fit: the unpaired Student *t* test, the Mann-Whitney *U* test, a 1-way analysis of variance, or Kruskal-Wallis. SVT-free survival curves were expressed using Kaplan-Meier estimates according to the four PALS quartiles. Univariable Cox-regression analysis was conducted to determine the hazard ratio of different parameters on a 5-year incidence of atrial arrhythmia. Patients with the lowest PALS quartile were compared to the other patients using a binary variable. To estimate the associations between parameters of interest and the response variable while adjusting for confounders, multivariable Cox-regression models were conducted using previously identified factors for supraventricular arrhythmias in addition to strain measures (age, LVMI, LAVI_max_ and E/e’) [11, 12]. The degree of agreement of LA strain measurements (PALS and PACS) was evaluated in 20 randomly selected patients, measured twice by CN and once by a second observer (AP). We applied the Bland-Altman method (mean difference, 95% confidence interval) and calculated the intra-class correlation coefficient. A two-tailed *p-*value of ≤ 0.05 was considered statistically significant. Statistical analyses were performed using IBM SPSS Statistics for Macintosh, version 28.0 (IBM Corp., Armonk, N.Y., USA).

## Results

### Patient selection and characteristics

**Fig. 2:**
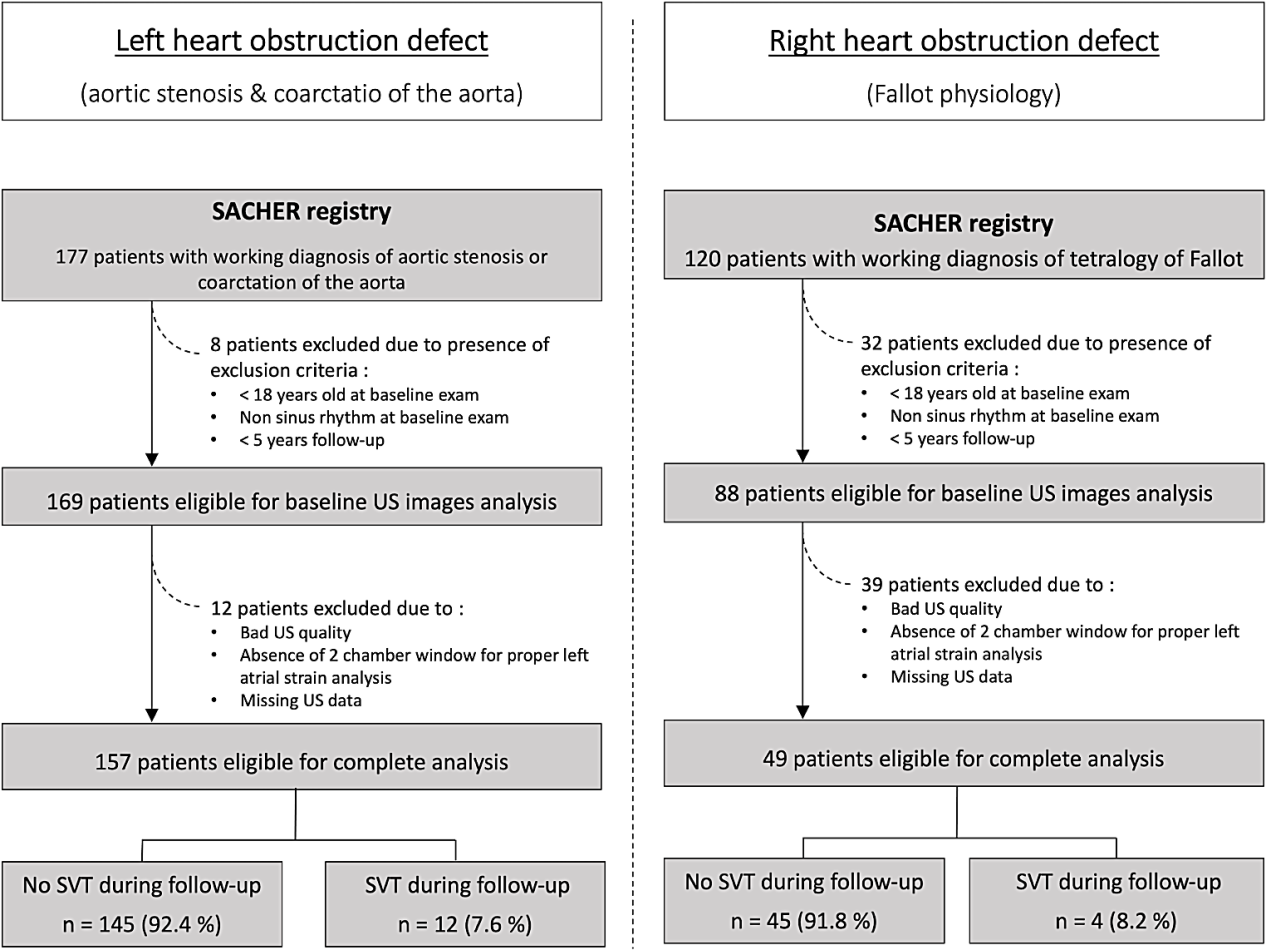
Of the 297 patients selected from the SACHER registry with aortic stenosis, coarctation of the aorta or Fallot physiology, 206 patients with sinus rhythm and adequate LA echocardiographic 2-chamber window were analyzed. During the 5-year follow-up, 16 patients (7.8%) developed atrial arrhythmia

Of the 297 patients screened, 206 fulfilled the selection criteria (selection process illustrated in Fig. 2). A total of 157 (76%) patients had left heart disease and 49 (24%) had a Fallot physiology. The median age at baseline was 29 years (IQR 22–41). 30% of the patients were female, and the median body mass index was 24.0 kg/m^2^ (IQR 21.8–26.3). Hypertension was present in 28% of the participants and 1% suffered from diabetes. Overall, 150 (73%) patients had undergone an intervention in the past, and the median time since the main repair was 19 years (IQR 8–27). Fifteen patients (7%) had a previous history of SVT. These patients were significantly older ( 52 ± 14 years vs. 32 ± 13 years, *p* < 0.001).

### Echocardiography

On transthoracic echocardiography, the mean left ventricular ejection fraction was 61 ± 7%. The median left ventricular mass index was 104 g/m^2^ (IQR 80–125) and 70 (34%) patients had LV hypertrophy. The median left atrial maximal and minimal volumes index (LAVI_max_, LAVI_min_) was 25 ml/m^2^ (IQR 21–32) and 10 ml/m^2^ (IQR 8–13), respectively. Overall, 37 patients (18%) had a dilated left atrium. The left atrial strain analyses yielded a median PALS of 40.3% (IQR 34.6–47.2%) and a median PACS of 16.9% (IQR 13.0–21.9%). In univariable analysis, higher PALS values were associated with lower LAVI_max_ (r = – 0.314, p < 0.001) and less advanced diastolic dysfunction according to E/e’ (*r* = – 0.235, *p* = 0.001). Table 1 provides detailed baseline clinical and echocardiographic characteristics of the patients in each group (left or right defect), including left atrial strain analyses.

**Table 1:** Baseline clinical characteristics

	Left heart defect	Right heart defect	
	*N* = 157 (76%)	*N* = 49 (24%)	*p* Value
**Patient characteristics**			
Age, *years*	28 (22 – 42)	29 (23 – 41)	0.69
Female	45/157 (29%)	16/49 (33%)	0.60
BMI, *kg/m*^*2*^	24.8 ± 3.9	23.7 ± 3.6	0.11
Systolic blood pressure, *mm Hg*	128 ± 18	124 ± 14	0.15
Diastolic blood pressure, *mm Hg*	74 ± 10	73 ± 11	0.60
Heart rate, *bpm*	70 ± 12	70 ± 10	0.83
NYHA functional class			0.046 *
I	146/157 (93.0%)	39/49 (79.6%)	
II	10/157 (6.4%)	10/49 (20.4%)	
III	1/157 (0.6%)	0/49 (0%)	
Hypertension	52/157 (33.1%)	5/49 (10.2%)	< 0.001 *
Previous heart surgery	101/157 (64.3%)	49/49 (100%)	< 0.001 *
Time since repair, *years*	13.6 (4.9 – 20.4)	24.8 (20.0 – 35.9)	< 0.001 *
Time since last surgery, *years*	6.2 (2.4 – 15.5)	7.3 (3.3 – 18.4)	0.20
**Defect**			
Left heart malformation	157		
Aortic valve stenosis	103/157 (66%)		
Coarctation of the aorta	54/157 (34%)		
Right heart malformation		49	
Tetralogy of Fallot		40/49 (82%)	
Double outlet right ventricle		7/49 (14%)	
Pulmonal atresia		2/49 (4%)	
**Imaging characteristics**			
***Left ventricle (echocardiography)***			
LV internal diastolic diameter, *mm*	50 ± 6	48 ± 7	0.10
LV mass index, *g/m*^*2*^	109 (84 – 133)	83 (75 – 112)	0.004 *
LV ejection fraction (Simpson, Biplane), *%*	62 ± 6	58 ± 8	0.16
e’ septal, *cm/s*	8.9 ± 2.2	8.8 ± 3.2	0.79
E/e’	9.2 (7.5 – 11.9)	10.2 (7.6 – 14.4)	0.27
E/A	1.5 ± 0.5	1.7 ± 0.6	0.08
***Left atrium (echocardiography)***			
LA max diameter in PLAX, *mm*	36 ± 7	38 ± 6	0.06
LA maximal volume index, *ml/m*^*2*^	25 (21 – 32)	24 (20 – 33)	0.50
LA minimal volume index, *ml/m*^*2*^	10 (8 – 13)	10 (8 – 13)	0.69
LA emptying fraction, *%*	59 (53 – 68)	61 (48 – 67)	0.33
LA expansion index, *%*	145 (112 – 213)	154 (93 – 208)	0.55
PALS, *%*	40.4 (34.6 – 46.5)	40.2 (34.3 – 48.6)	0.56
PACS, *%*	16.4 (12.7 – 21.2)	18.6 (14.0 – 22.6)	0.14
***Right ventricle (echocardiography)***			
TAPSE, *mm*	21 ± 5	16 ± 4	< 0.001 *
DTI, *cm/s*	12 ± 3	9 ± 2	< 0.001 *
Impaired RV function, *n (%)*	4/157 (3%)	23/49 (47%)	< 0.001 *
***Valve characteristics (echocardiography)***			
Aortic valve peak gradient, *mm Hg*	13 (8 – 23)	5 (4 – 6)	< 0.001 *
Aortic valve mean gradient, *mm Hg*	7 (5 – 13)	3 (2 – 4)	< 0.001 *
AVAi, *cm*^*2*^*/m*^*2*^	1.0 (0.8 – 1.4), (*N* = 113)		
Pulmonal valve peak gradient, *mm Hg*		20 (11 – 30)	
***CMR right ventricle***			
RV EDVi, *ml/m*^*2*^		131 (108 – 144)	
RV ejection fraction, *%*		49 (44 – 55)	
Pulmonal valve reflux fraction, *%*		50 ± 8	

Values are median (interquartile range) or n (%). Statistically significant differences between the two groups are represented with a *

A = late diastolic mitral inflow velocity; AVAi = aortic valve area indexed; BMI = body mass index; DT = E-wave deceleration time; DTI = tissue doppler imaging; E = peak early diastolic mitral flow velocity; e’ = peak early diastolic mitral annular velocity; EDVi = end diastolic volume indexed; LA = left atrial; LV = left ventricular; NYHA = New York Heart Association Functional Classification; PALS = peak atrial longitudinal strain; PACS = peak atrial contraction strain; PLAX = parasternal long axis US window; RV = right ventricular; TAPSE = tricuspid annular plane systolic excursion

### Atrial arrhythmia

During a median follow-up of 6.2 years (IQR 5.6–7.3), 16 patients (8%) developed sustained atrial arrhythmias after a mean follow-up of 3.3 ± 2.5 years. Twelve of them (75%) suffered from atrial fibrillation and four (25%) had intra-atrial re-entry tachycardia. Seven of these patients (44%) had a new onset of SVT, while nine (56%) had a recurrence of atrial arrhythmia. There was no significant difference between the prevalence of arrhythmias in patients with a left or right heart defect (7.6% vs. 8.1%, respectively; p = 0.906). Patients who developed SVT were older and had larger left atrial volumes in terms of LAVI_max_ and LAVI_min_, a higher LVMI, a higher likelihood to have increased left ventricular filling pressures expressed by the E/e’ ratio, and lower PALS. Table 2 compares the baseline characteristic parameters between SVT and non-SVT subgroups in detail.

**Table 2:** Baseline clinical characteristics in *n* = 206 patients with and without SVT

	With SVT	Without SVT	
	*N* = 16 (8%)	*N* = 190 (92%)	*p* Value
**Patient characteristics**			
Age, *years*	51 (33 – 58)	28 (22 – 38)	< 0.001*
Female	1/16 (6%)	60/190 (32%)	0.002*
BMI, *kg/m*^*2*^	25.7 ± 3.5	24.4 ± 3.9	0.20
Systolic blood pressure, *mm Hg*	132 ± 11	127 ± 17	0.29
Diastolic blood pressure, *mm Hg*	76 ± 6	73 ± 10	0.37
Heart rate, *bpm*	67 ± 9	70 ± 12	0.27
NYHA functional class			0.68
I	14/16 (88%)	171 (90%)	
II	2/16 (12%)	18 (10%)	
III	0/16 (0%)	1 (1%)	
Hypertension	7/16 (44%)	50 (26%)	0.20
Past heart surgery	11/16 (69%)	139 (73%)	0.71
Time since repair, *years*	36.9 (15.9 – 50.2)	18.6 (7.5 – 24.7)	< 0.001*
Time since last surgery, *years*	3.6 (0.9 – 8.9)	7.2 (2.9 – 16.9)	0.17
Number of heart surgery	2 (0 – 5)	1 (0 – 2)	0.001*
**Patient working diagnosis**			
Left heart malformation	12/16 (75%)	145/190 (76%)	
Right heart malformation	4/16 (25%)	45/190 (24%)	
**Echocardiographic characteristics**			
***Left ventricle***			
LV internal diastolic diameter, *mm*	54 ± 6	49 ± 7	0.013*
LV mass index, *g/m*^*2*^	139 (115 – 159)	100 (79 – 124)	< 0.001*
RWT	0.46 ± 0.14	0.42 ± 0.11	0.19
LV ejection fraction (Simpson, Biplane), *%*	60 ± 7	61 ± 7	0.80
e’ septal, *cm/s*	5.3 (3.9 – 8.6)	8.9 (7.5 – 10.5)	< 0.001*
E/e’	16.1 (8.1 – 26.7)	9.4 (7.5 – 11.6)	0.024*
E/A	1.5 ± 0.4	1.6 ± 0.6	0.67
DT, *ms*	204 (158 – 241)	190 (155 – 229)	0.48
***Left atrium***			
LA max diameter in PLAX, *mm*	44 ± 7	35 ± 6	< 0.001*
LA maximal volume index, *ml/m*^*2*^	33 (29 – 49)	24 (20 – 31)	0.043*
LA minimal volume index, *ml/m*^*2*^	16 (11 – 29)	10 (8 – 12)	0.009*
LA emptying fraction, *%*	49 (39 – 62)	60 (53 – 68)	0.020*
LA expansion index, *%*	95 (63 – 160)	150 (114 – 212)	0.015*
PALS, *%*	27.3 (22.2 – 33.3)	41.1 (35.2 – 48.0)	< 0.001*
PACS, *%*	14.5 (12.3 – 17.9)	17.0 (13.0 – 22.2)	0.23
***Right ventricle***			
TAPSE, *mm*	18 ± 5	20 ± 5	0.27
DTI, *cm/s*	11 ± 3	12 ± 3	0.16
Impaired RV function, *n (%)*	5 (31%)	22 (12%)	0.025*

Values are median (interquartile range) or n (%). Statistically significant differences between the two groups are represented with a *

A = late diastolic mitral inflow velocity; AVAi = aortic valve area indexed; BMI = body mass index; DT = E-wave deceleration time; DTI = tissue doppler imaging; E = peak early diastolic mitral flow velocity; e’ = peak early diastolic mitral annular velocity; LA = left atrial; LV = left ventricular; NYHA = New York Heart Association Functional Classification; PALS = peak atrial longitudinal strain; PACS = peak atrial contraction strain; PLAX = parasternal long axis US window; RV = right ventricular; RWT = relative wall thickness; SVT = supraventricular tachyarrhythmia; TAPSE = tricuspid annular plane systolic excursion

### Parameters associated with new onset of SVT

**Fig. 3:**
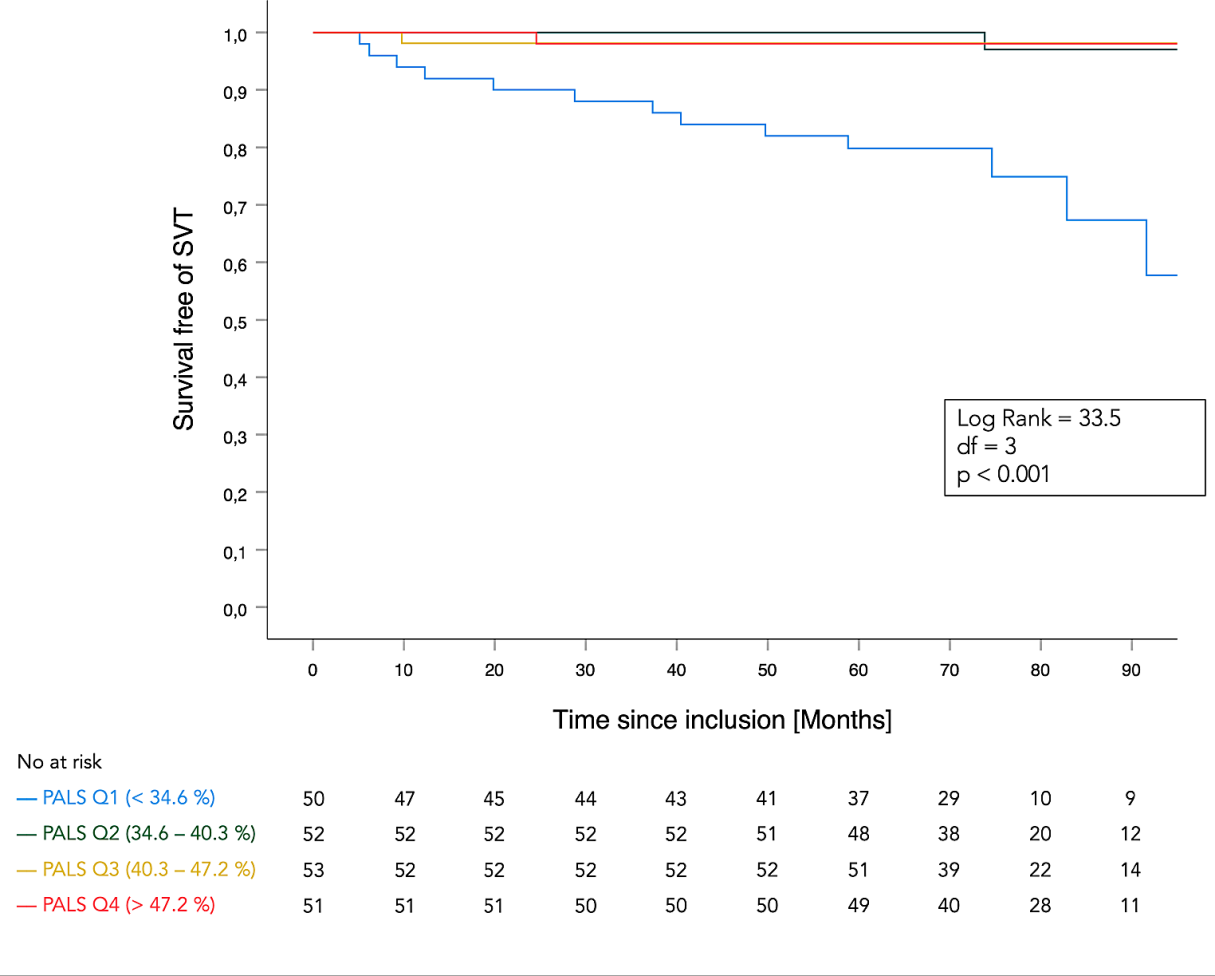
Shows the Kaplan-Meier curve of survival-free of SVT using PALS quartiles. Patients with the lowest quartile value for PALS (< 34.6%) had significantly higher risk of developing SVT during the 5-year follow-up

By Cox regression analysis, patients in the lowest quartile for PALS (PALS < 34.6%) had a 15.9 hazard ratio (95% CI: 4.5 to 56.0, p < 0.001) of SVT compared to patients within the top three quartiles, using a binary variable approach. The Kaplan-Meier estimate for survival-free of SVT as a function of PALS quartiles yielded a significant difference (p < 0.001 in the Log-rank test) between the first and the remaining quartiles (Fig. 3). A PALS value < 34.6% (cut-off between the first and second PALS quartile) was associated with an increased risk of atrial arrhythmia in both left and right heart defect patients. In univariate Cox regression models, age at baseline, LVMI, echocardiographic parameters for estimation of LV diastolic function (E/e’ and e’), and LA parameters except for PACS were all significantly associated with SVT. In multivariable Cox regression models with age, LVMI, LAVI_max_, E/e’ and PALS as factors associated with arrhythmias, the association between reduced PALS and SVT risk remained robust, independently from the other parameters (Table 3).

**Table 3:** Uni- and multivariable predictors of SVT (Cox Regression model)

	*p* Value	HR	95% CI
**Patient characteristics**			
Age, *y*	< 0.001	1.07	1.04 – 1.10
Male	0.07	6.52	0.86 – 49.4
BMI, *kg/m*^*2*^	0.21	1.07	0.96 – 1.19
Hypertension	0.14	2.11	0.79 – 5.68
Past heart surgery	0.73	0.86	0.29 – 2.38
Time since repair, *years*	< 0.001	1.08	1.03 – 1.13
Number of heart surgery	0.002	1.37	1.12 – 1.68
Patient diagnosis	0.91	1.07	0.35 – 3.32
**Echocardiographic characteristics**			
***Left ventricle***			
LV internal diastolic diameter, *mm*	0.003	1.11	1.04 – 1.19
LV mass index, *g/m*^*2*^	< 0.001	1.02	1.01 – 1.03
LV ejection fraction (Simpson, Biplane), *%*	0.63	0.99	0.99 – 1.04
e’ septal, *cm/s*	< 0.001	0.50	0.35 – 0.73
E/e’	< 0.001	1.24	1.14 – 1.35
***Left atrium***			
LA max diameter in PLAX, *mm*	< 0.001	1.18	1.10 – 1.27
LA maximal volume index, *ml/m*^*2*^	< 0.001	1.04	1.03 – 1.06
LA minimal volume index, *ml/m*^*2*^	< 0.001	1.12	1.08 – 1.16
LA emptying fraction, *%*	< 0.001	0.93	0.90 – 0.96
LA expansion index, *%*	0.010	0.28	0.11 – 0.74
PALS, *%*	< 0.001	0.84	0.79 – 0.89
PACS, *%*	0.22	0.95	0.88 – 1.03
PALS *(first vs. other quartiles)*	< 0.001	15.91	4.52 – 56.02
***Right ventricle characteristics***			
TAPSE, *mm*	0.34	0.96	0.88 – 1.05
DTI, *cm/s*	0.16	0.89	0.75 – 1.05
Impaired RV function, *n (%)*	0.037	3.09	1.07 – 8.90
**Tri-variate Cox-regression analyses**			
PALS	< 0.001	0.86	0.79 – 0.94
Age	0.080	1.03	1.00 – 1.07
LV mass index	0.004	1.01	1.00 – 1.02
PALS	< 0.001	0.88	0.82 – 0.94
Age	0.041	1.04	1.00 – 1.07
LAVI_max_	0.182	1.02	0.99 – 1.05
PALS	0.024	0.88	0.78 – 0.98
Age	0.081	1.04	0.99 – 1.09
E/e’	0.009	1.10	1.02 – 1.17

BMI = body mass index; CI = confidence interval; E = peak early diastolic mitral flow velocity; e’ = peak early diastolic mitral annular velocity; DTI = tissue doppler imaging; HR = hazard ratio; LA = left atrial; LV = left ventricular; PALS = peak atrial longitudinal strain; PACS = peak atrial contraction strain; PLAX = parasternal long axis US window; RV = right ventricular; TAPSE = tricuspid annular plane systolic excursion

In a post-hoc analysis and after excluding patients with history of prior SVT (15/206 patients), reduced PALS (< 34.6%) remained significantly associated with onset of SVT during follow-up with a 10.1 hazard ratio (95% CI: 2.0 to 52.4, *p* = 0.006).

### Reproducibility

The intraobserver and interobserver variability as described with the intraclass correlation coefficient was 0.88 and 0.80 for PALS, and 0.95 and 0.85 for PACS, respectively. Mean difference and 95% confidence interval for intraobserver and interobserver comparison was – 0.01% (–2.59 to 2.57) and – 2.82% (–7.032 to 1.39) for PALS, and – 0.98% (–2.24 to 0.27) and 0.16% (–2.01 to 2.33) for PACS, respectively.

## Discussion

This retrospective cohort study assessed the association of atrial strain parameters with the development of SVT in the ACHD population. Our results showed that PALS, a measure of the LA reservoir function, was independently associated with these SVTs, both in patients with left and right heart defects. In contrast, PACS as a measure of LA booster function was not significantly associated with the development of atrial arrhythmias. Finally, we were able to confirm that there was a correlation between older age, more cardiac surgeries, and larger LA volumes, and the development of SVT in the ACHD population.

Previous studies showing that LA strain is a predictor of atrial arrhythmias have focused on patients with acquired heart disease. To our knowledge, this study was the first to assess the association between atrial strain and SVT in the ACHD population. There are important differences in baseline characteristics between patients with acquired versus congenital heart disease: ACHD patients are usually younger than patients with acquired heart disease and have fewer co-morbidities, but more atrial sutures lines from previous cardiac surgeries. Hence results from patients with acquired heart disease may not apply to ACHD cohorts.

### SVT and its predictors in congenital heart disease

In the aging ACHD population, arrhythmias are the most common long-term complication and account for 31% of all hospitalisations [1, 17–19]. For these patients, SVT doubles the risk of heart failure or stroke with a hazard ratio of 2.21, and mortality is increased by 50% [6].

The clinical predictors of atrial arrhythmias differ for ACHD patients, compared to the general population. Unique factors, such as heart defect complexity, must be examined. As in previous studies [4, 20, 21], we demonstrated a correlation between the age and the number of heart surgical procedures, and the development of atrial arrhythmias. In addition, the atrial volume has previously been used to assess atrial overload and structural remodeling, which is a substrate for atrial tachyarrhythmias [10]. Our study supports these findings, with larger LAVI_min_ and LAVI_max_ being associated with development of SVT. Furthermore, we found a significant association between LVMI and echocardiographic measurements of left diastolic function (e’ septal and E/e’ ratio), and atrial arrhythmias. This may illustrate a similar pathological pathway to that of patients with acquired HFpEF. In both situations, the cause appears to be a long-term pressure overload, leading to LA enlargement, remodeling, and probably a decrease in the left atrial function.

By studying left atrial strain, our current study explores additional echocardiography parameters than the traditional markers of diastolic filling pressure and left atrial volume. Patients in the lowest PALS quartile were more likely to develop SVT in the follow-up period with a 16-fold higher hazard ratio in univariate analysis. In different multivariable analysis models with PALS, age, LVMI, LAVI_max_ and E/e’, PALS had an independent and incremental predictive value for atrial arrhythmia. This atrial strain parameter yields information about the function of the left atrium, which differs from the structural information provided by atrial volumes. This has already been documented in patients with acquired heart disease [11–13], including heart failure with preserved ejection fraction (HFpEF) or rheumatic mitral stenosis.

We noted that our PALS threshold, ≤ 34.6%, is notably higher than those stated in the three articles mentioned above (ranging from 17 to 29%). This may suggest that ACHD patients with a younger age have still more preserved atrial function than the elder cohorts with acquired heart disease and a subtle decline in this function may have wider implications for them.

### Clinical implications and future perspectives

Echocardiographic atrial strain analyses with measurement of PALS allowed us to identify ACHD patients at risk of developing atrial arrhythmias. Using PALS as an early sensor of atrial function decline may enable us to address subclinical atrial deterioration by reducing atrial pressure overload at an earlier stage and prevent the occurrence of irreversible remodeling. Previous studies have shown that, in the general population, it is possible to reverse atrial remodeling if the stressors that lead to functional and structural alteration of the atria are addressed early [10, 22, 23]. Aggressive lifestyle modification and pharmacotherapy appear to play an important role [23, 24]. One can also conceive the beneficial impact of early reintervention to improve hemodynamic abnormalities in ACHD patients. Further studies need to address these hypotheses in the ACHD population.

One can speculate on the usefulness of preventive thromboprophylaxis in the ACHD patient at high risk of developing SVT and in sinus rhythm. However, it is still unclear how to assess thromboembolic risk in this population [25]. Future randomized trials could examine the potential for beneficial thromboprophylaxis in ACHD patients in sinus rhythm with a high risk of SVT.

### Limitation of our study

Firstly, this study is a retrospective analysis of a cohort and therefore remains observational. Some confounding factors could hence have affected our results. Secondly, we excluded patients with an inappropriate image quality of the left atrium and this selection could have led to another potential bias. Thirdly, the difference in strain analysis software may make our results less generalizable, although it has been shown that there is no significant impact of software on LA strain results [26]. Fourth, our cohort size did not meet the initially calculated sample size based on conservative assumptions for the event rate. As our observed event rate was higher than assumed, we are able to show a significant correlation between PALS and arrhythmias. With a relatively small number of patients developing SVT during follow-up, we intentionally limited the number of parameters in the multivariable analysis on their predictive impact on the development of SVT, to avoid potential collinearity. Therefore we can not exclude the predictive value of additional parameters not studied in the current model. Fifth, we chose a SVT-free interval of 6 months as inclusion criteria. This may have led to the inclusion of patients at higher risk of developing SVT during follow-up, and to an overestimation of the event rate. Sixth, systematic SVT assessment during follow-up could not be conducted because of the retrospective nature of this study. This could have led to an underestimation of the event rate. Finally, we only focused our study on a small number of congenital heart diseases. Hence, our findings may not be fully applicable to the entire ACHD population.

## Conclusion

Lower PALS is significantly associated with the development of SVT in patients with congenital heart defects (aortic stenosis, coarctation of the aorta, and Fallot’s physiology) with an incremental value to previously established echocardiographic and clinical predictors. Atrial functional information from PALS combined with atrial structural data and other clinical values may allow for the identification and stratification of ACHD patients at risk of arrhythmia. Future studies are needed to refine the assessment of the risk of atrial arrhythmias in this population including left atrial strain measures.

## Data Availability

No datasets were generated or analysed during the current study.

## Abbreviations

ACHD: adult with congenital heart disease
ECG: echocardiogram
LA: left atrial
LAVI: left atrial volume index
LVMI: left ventricular mass index
LVOT: left ventricular outflow tract
PACS: peak atrial contraction strain
PALS: peak atrial longitudinal strain
SACHER: swiss adult with congenital heart disease registry
SVT: supraventricular tachyarrhythmia
